# Heterogeneous responses of wetland vegetation to climate change in the Amur River basin characterized by normalized difference vegetation index from 1982 to 2020

**DOI:** 10.3389/fpls.2023.1290843

**Published:** 2023-11-01

**Authors:** Zihan Xing, Xiaoyan Li, Dehua Mao, Ling Luo, Zongming Wang

**Affiliations:** ^1^ College of Earth Sciences, Jilin University, Changchun, China; ^2^ State Key Laboratory of Black Soils Conservation and Utilization, Northeast Institute of Geography and Agroecology, Chinese Academy of Sciences, Changchun, China; ^3^ National Earth System Science Data Center, Beijing, China

**Keywords:** wetlands, vegetation change, NDVI, climate change, Amur River basin

## Abstract

Climate change affects wetland vegetation dramatically in mid- and high- latitudes, especially in the Amur River basin (ARB), straddling three countries and distributing abundance wetlands. In this study, spatiotemporal changes in average normalized difference vegetation index (NDVI) of wetland during the annual growing season were examined in the ARB from 1982 to 2020, and the responses of wetland vegetation to climatic change (temperature and precipitation) in different countries, geographic gradients, and time periods were analyzed by correlation analysis. The NDVI of wetland in the ARB increased significantly (*p* < 0.01) at the rate of 0.023 per decade from 1982 to 2020, and the NDVI on the Russian side (0.03 per decade) increased faster than that on the Chinese side (0.02 per decade). The NDVI of wetland was significantly positively correlated with daily mean temperature (*p* < 0.05, *r* = 0.701) and negatively correlated with precipitation, although the correlation was not significant (*p* > 0.05, *r* = −0.12). However, the asymmetric effects of diurnal warming on wetland vegetation were weak in the ARB. Correlations between the NDVI of wetland and climatic factors were zonal in latitudinal and longitudinal directions, and 49°N and 130°E were the points for a shift between increasing and decreasing correlation coefficients, closely related to the climatic zone. Under climate warming scenarios, the NDVI of wetland is predicted to continue to increase until 2080. The findings of this study are expected to deepen the understanding on response of wetland ecosystem to global change and promote regional wetland ecological protection.

## 1 Introduction

Vegetation responded dramatically to global climate change in terrestrial ecosystems (Cramer et al., 2001). Wetlands, one of the most important ecosystems, are critical to biodiversity conservation, carbon sequestration, and hydrological and climate regulation (Mao et al., 2021; Salimi et al., 2021). In addition to various ecological functions, wetlands also have certain socioeconomic and cultural values, such as recreation, tourism, and scientific research (Pedersen et al., 2019). However, wetlands are the most vulnerable ecosystems and changes in hydrology, soils, climate, and anthropogenic disturbances all affect the ecological stability of wetlands (Bansal et al., 2019). Wetland vegetation, the main component of wetland ecosystems, is particularly susceptible to dramatic global climate change (Erwin, 2009). Compared with vegetation in other ecosystems, the unique growth environment of wetland vegetation results in obvious differences in the responses of wetland vegetation to climate change (Pang et al., 2017; Boulanger et al., 2018; Shen et al., 2022). Therefore, understanding how wetland vegetation changes in response to climatic change is essential for the adaptive management and conservation of wetlands.

Climate change affects the growth of wetland vegetation especially at the mid- and high-latitudes (Peng et al., 2020; Yuan et al., 2020). The global climate is undergoing a change characterized mainly by warming. Specifically, warming climate has advanced spring phenology and delayed autumn phenology, thereby extending the growing season of wetland vegetation (Herfindal et al., 2012; Crabbe et al., 2016). Although increases in precipitation can increase photosynthetic activity and promote wetland vegetation growth (Peng et al., 2013; Chen et al., 2020), seasonal increases in precipitation can adversely affect the reproduction of wetland vegetation by raising water levels and submerging vegetation (Bardecki, 1991). In previous studies, changes in wetland vegetation in response to climate change in several regions of the Northern Hemisphere were examined by using vegetation indices (Herfindal et al., 2012; Liu et al., 2022; Ren et al., 2022), such as normalized difference vegetation index (NDVI) and enhanced vegetation index. However, due to geographical differences, global climate change is spatially heterogeneous, and different climatic factors have varying impacts on wetland vegetation (Shen et al., 2022). Because of the heterogeneous response of wetland vegetation to climate change, additional research is needed that focuses on detailed analyses in order to develop adaptive management and future conservation strategies, especially in mid- and high latitudes.

As an important indicator of wetland vegetation health and growth, NDVI has been widely used in regional monitoring of changes in wetland vegetation and vegetation feedback on regional climate (Di et al., 1994; Meneses-Tovar, 2011; Wei et al., 2022). Because climate change is a long-term process, applying long time series data to investigate the responses of wetland vegetation to climate change can benefit understanding of processes of change in wetland vegetation and future wetland adaptive management (Hasselmann et al., 2003). Currently, data from sensors such as the Advanced Very High Resolution Radiometer (AVHRR) onboard the National Oceanic and Atmospheric Administration (NOAA) and Moderate Resolution Imaging Spectroradiometer (MODIS) are widely used in analyzing the NDVI of wetland dynamics (Fensholt and Sandholt, 2005; Albarakat and Lakshmi, 2019). Due to the limitation of data sources, in previous studies based on MODIS NDVI, changes in wetland vegetation in response to climate change were analyzed from 2000. However, it is difficult to fully understand longer term changes in wetland vegetation on the basis of those studies (Wang et al., 2020; Chen et al., 2023). Continuous long time series of NDVI can accurately reflect long-term trends in changes in wetland vegetation and abrupt changes (Guo et al., 2021). The longest time series is the NOAA Climate Data Record (CDR) of AVHRR NDVI data set, which is therefore the most suitable data source to analyze wetland vegetation change at both large scale and long time series (Peng et al., 2012). Moreover, with advantages such as free, huge computing power and rapid batch processing of data, the Google Earth Engine (GEE) cloud platform allows acquisition and rapid processing of large-scale long time series NDVI data sets. (Liu et al., 2018; Phan et al., 2020). Therefore, the use of NOAA CDR of AVHRR NDVI data set and GEE processing platform provides a possibility for long-term large-scale wetland analysis.

The Amur River basin (ARB), the world’s ninth largest river basin, is an area of wetland concentration in mid- and high-latitude zones, where wetlands are particularly sensitive to global climate change (Wang et al., 2019; Han et al., 2020). Compared with North America and Europe in the same latitudes, wetland research is limited in the ARB (Wang Y. et al., 2020; Mao et al., 2021). China has higher temperatures and less precipitation, but more anthropogenic activity than Russia, and as a result, responses of wetland vegetation to climate change also differ (Chu et al., 2019). Nevertheless, there is a lack of comparative studies that explore changes in wetland vegetation and its response to climate change on Chinese and Russian sides of the ARB. A comparative analysis of environments in China and Russia would help provide a more comprehensive understanding of wetland changes in the ARB and support wetland conservation policies in both countries. In addition, temperature data show faster warming during the night than during the day in the past few decades in the ARB (Peng et al., 2013), and asymmetric effects of monthly average daily maximum temperature (TMX) and monthly average daily minimum temperature (TMN) on the NDVI of wetland were observed on Songnen (Wang Y. et al., 2020) and Sanjiang plains (Liu et al., 2022), which are part of the basin. However, the asymmetric effects differed on these plains. The NDVI of wetland was negatively correlated with TMX and positively correlated with TMN during the growing season on Songnen Plain (Wang Y. et al., 2020), but increasing TMN was more effective in promoting wetland vegetation growth than increasing TMX on Sanjiang Plain (Liu et al., 2022). Until recently, previous studies have been limited to small-scale analyses, and it has remained unclear whether there are long-term asymmetric effects of diurnal warming on wetland vegetation in the entire ARB. To accurately predict future changes in wetland vegetation, it is also necessary to explore the responses of wetland vegetation to changes in diurnal temperatures.

In this paper, therefore, the heterogeneous responses of wetland vegetation to climate factors in the ARB were examined from 1982 to 2020. The specific objectives were to (1) explore spatiotemporal changes in wetland vegetation in the ARB during the growing season from 1982 to 2020; (2) examine the responses of wetland vegetation to climate change in different countries, geographic gradients, and time periods; and (3) predict the effects of future climate change on wetland vegetation in the ARB. The study is expected to provide theoretical support for the conservation and restoration of wetlands.

## 2 Materials and methods

### 2.1 Study area

**Figure 1:**
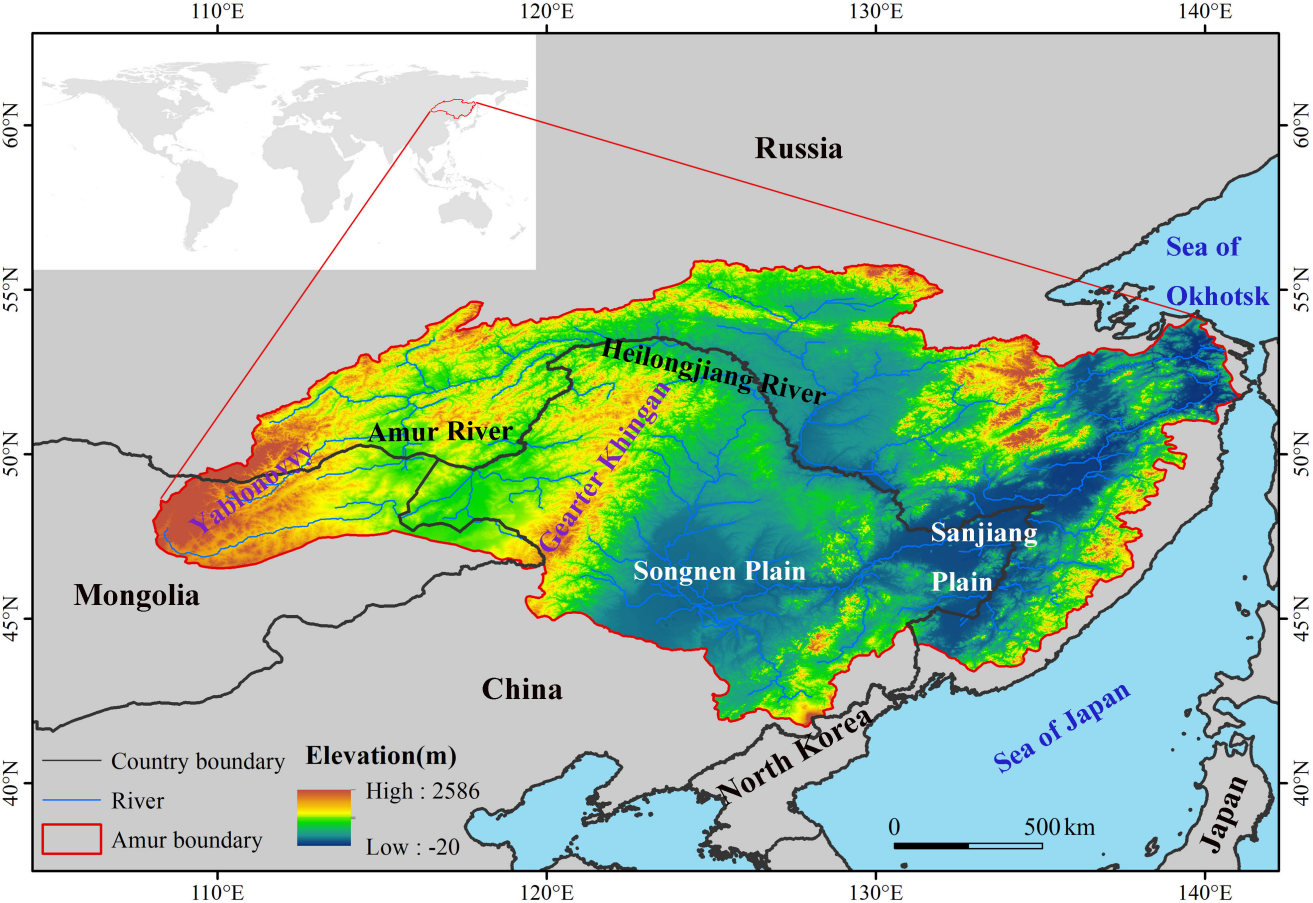
Location of the Amur River basin and its spatial terrain pattern.

The ARB spans latitudes from 41.72°N to 55.90°N and longitudes from 108.05°E to 141.13°E. It is a transboundary region of three countries (**Figure 1**), with 48% of the basin in Russia, 43% in China, and 9% in Mongolia, and a total area of 2.08 million km^2^ (Mao et al., 2021). The Greater Khingan Mountains divide the ARB into two relatively different climates. The western ARB is relatively dry, with annual precipitation of only 250 mm–400 mm, whereas the eastern ARB is relatively wet, with annual precipitation of 400 mm–700 mm. The ARB passes through humid, semi-humid and semi-arid zones, with boundaries between the three zones at 130°E and 120°E, respectively, which is continental monsoon climate. Annual average temperature is between −8°C and 6°C, decreasing gradually from south to north, with clear spatial variability. The basin straddles two temperature zones from south to north, mid-temperate and frigid-temperate zones, with the boundary between the two zones at 50°N. There are many rivers and developed water systems that crisscross the basin, interacting to form the Songnen and Sanjiang plains in the east, with lower elevations than those of the mountains in the east. The ARB has a large wetland area that is relatively concentrated in the Greater Khingan Mountains and Songnen and Sanjiang plains. The main wetland vegetation types common in this basin are Typha orientalis, Phragmites australis, and Scirpus triqueter, and the water level of suitable wetlands is gradually declining. The wetlands provide important resting places for multitudes of migratory waterfowl on the East Asian–Australasia flyway (Jia et al., 2020). The total wetland area of the ARB is 0.17 million km^2^, accounting for 8.1% of the ARB (Yang et al., 2020). However, there were fewer wetlands located in Mongolia, and almost no unchanged wetlands, extracted by the wetland data, were located in Mongolia, so we mainly analyzed the changes of wetlands in the Chinese and Russian sides.

### 2.2 Data source

#### 2.2.1 NDVI data

The NOAA CDR of AVHRR NDVI version 5 was used in this study. The resolution was 0.05°, and the interval was 1 day. Free images were collected in the growing season (May–September) from 1982 to 2020 on the GEE cloud platform (https://earthengine.google.com/). Monthly NDVI data were obtained by the maximum value composite method, which chose the largest value of each pixel in the multitemporal data to reduce the interference of atmospheric and solar zenith angle (Wang et al., 2005), and projection processing and clipping were processed on GEE.

#### 2.2.2 Climatic data

Climatic data were acquired from the monthly gridded Climatic Research Unit Time-Series Data version 4.05(CRU TS 4.05, available at https://crudata.uea.ac.uk/cru/data/hrg/). The CRU TS data were produced by the CRU at the University of East Anglia with a resolution of 0.5° and a long time series from 1901 to 2020. The data have been widely used in studies on climate change and on vegetation growth (Chu et al., 2019; Meng et al., 2019). In this study, four types of climatic factors acquired from CRU TS 4.05 were used to analyze the effect of climate on wetland vegetation change, including daily mean temperature (TMP), TMX, TMN, and precipitation (PRE). To study asymmetric effects of diurnal warming on wetland vegetation in the ARB, TMX and TMN were used to represent daytime and nighttime temperatures, respectively. To coordinate data sets, the spatial resolution of temperature and precipitation data was resampled to 0.05° by using the nearest neighbor method.

#### 2.2.3 Wetland data

Wetland distribution data were extracted from up-to-date land cover data in the ARB in 1980 and 2016 (Mao et al., 2020). Natural wetlands in this study were vegetated wetlands, including marsh, swamp, bog, and fen. Land cover data were classified from Landsat TM/OLI images by an object-based image analysis and hierarchical decision–trees classification method, including multi­resolution segmentation, designing place-based hierarchical decision trees, and rule-based object identification (Mao et al., 2020). The data were obtained from the Northeast branch of the National Earth System Science Data Center (http://wetland.igadc.cn/). Accuracy of the data was verified by field investigations and Google Earth images with overall classification accuracy values larger than 90% (Mao et al., 2021), which showed that the wetland data were reliable to explore the responses of wetland vegetation to changes. For the analysis of NDVI trends and its relations with climatic factors, wetlands existing both in 1980 and 2016 were extracted as unchanged wetlands from 1980 to 2016 in the ARB, with a total area of 0.12 million km^2^.

#### 2.2.4 Future climate data

The future temperature and precipitation data used in this study were produced from the CNRM–ESM2–1 global climate model of the Scenario Model Intercomparison Program (ScenarioMIP) under International Coupled Model Intercomparison Program Phase 6 (CMIP6, available at https://esgf-node.llnl.gov/search/cmip6/). The CNRM–ESM2–1 model provided by CMIP6 was derived from the French National Meteorological Center, with a spatial resolution of 1.4° × 1.4°. The combination scenarios of Shared Socioeconomic Pathways (SSPs) and Representative Concentration Pathways (RCPs) incorporates the impact of socioeconomic development (Su et al., 2021), which are widely used in regional climate change prediction (Yang et al., 2020; Ren et al., 2023). SSP1–RCP2.6 is a low-forcing scenario, and SSP5–RCP8.5 is a high-forcing scenario.

### 2.3 Methods

#### 2.3.1 Sen+Mann–Kendall method

In this study, we have used Sen+Mann–Kendall method based on the R 4.2.1 to analyze trends from 1982 to 2020 for the NDVI of wetland vegetation and four climatic factors (PRE, TMP, TMX, and TMN). Due to the strong resistance to measurement errors and abnormal data, the Sen+Mann–Kendall method has been increasingly used to analyze trends in long term series data for vegetation (Li et al., 2021; Wu et al., 2021), (Li et al., 2021).

Theil–Sen median trend analysis is a non-parametric statistical trend calculation method (Sen, 1968). The formula is as follows:


X(t)=Qt+B

where *X(t)* is the time series data of climate and NDVI, *Q* is the slope of the data, and B is a constant.


$$Q_{median}=Median\left(\frac{X_{j}-X_{i}}{j-i}\right)$$

where *Q_median_* is the median of *Q* sorted from smallest to largest. When *Q_median_* > 0, the climate or NDVI time series has an increasing trend, whereas when *Q_median_* < 0, a decreasing trend is indicated.

The Mann–Kendall trend test is a non-parametric statistical test method that determines the significance of trends (Kendall, 1948). The formula is as follows:


$$S=\sum_{i=1}^{n-1}\sum_{j=i+1}^{n}sgn\left(X_{j}-X_{i}\right)$$

where *n* is the length of the time series (39 years in this study), and *sgn(X_j_–X_i_)* is the sign function, defined as follows:


$$sgn\left(X_{j}-X_{i}\right)=\{\begin{array}{c} -1\;\;if\;X_{j}-X_{i}<0\\ \;\;0\;\;if\;X_{j}-X_{i}=0\\ \;\;1\;\;if\;X_{j}-X_{i}>0 \end{array}$$

For the time series data *X(t)*, the statistic *Z* is defined as follows:


$$Z=\{\begin{array}{ll} \frac{S+1}{\sqrt{Var\left(S\right)}} & S<0\\ \;0 & S=0\\ \frac{S-1}{\sqrt{Var\left(S\right)}} & S>0 \end{array}$$

where $Var\left(S\right)=\frac{n\left(n-1\right)\left(2n+5\right)}{18}$ . When *Z* > 0, the variable shows an upward trend; when *Z*< 0, the variable shows a downward trend. In addition, when |*Z*| > 1.96, the time series has significant variation at the level of 0.05 (Liu et al., 2019). According to the significance of trends, variations in NDVI trends were classified into three types: significant increase (slope > 0, *p* < 0.05), significant decrease (slope< 0, *p* < 0.05), and nonsignificant change (*p* > 0.05).

#### 2.3.2 Hurst exponent

We have used Hurst exponent to investigate whether trends of the NDVI of wetland vegetation was sustainable in the ARB from 1982 to 2020. The method was proposed by Hurst (1951) in the analysis of hydrological data and then improved by Mandelbrot and Wallis (1969). The method has been successfully applied in studies investigating vegetation changes (Jiang et al., 2017). The basic calculations are as follow:

1) Divide the time series {NDVI(τ)}(τ=1,2,⋯,n) into τ sub series *X*(*t*), for each series t=1,2,⋯,τ .

2) Define the mean sequence of the time series,


$$\bar{NDVI_{\left(\text{τ}\right)}}=\frac{1}{\text{τ}}\sum_{t=1}^{\text{τ}}NDVI_{\left(t\right)}$$

3) Calculate the accumulated deviation,


$$X_{\left(t\right)}=\sum_{t=1}^{t}\left(NDVI_{\left(t\right)}-\bar{NDVI_{\left(\text{τ}\right)}}\right)\;1\leq t\leq \text{τ}$$

4) Create the range sequence,


$$R\left(t\right)=\underset{1\leq t\leq \text{τ}}{\max}X_{\left(t,\text{τ}\right)}-\underset{1\leq t\leq \text{τ}}{\min}X_{\left(t,\text{τ}\right)}\text{ τ}=1,2,\cdots ,n$$

5) Create the standard deviation sequence,


$$S_{\left(\text{τ}\right)}=\sqrt{\frac{1}{\tau }\sum_{t=1}^{\tau }{\left(NDVI_{\left(t\right)}-NDVI_{\left(\text{τ}\right)}\right)}^{2}}\text{ τ}=1,2,\cdots ,n$$

6) Calculate the Hurst exponent


$$\frac{R_{\left(\text{τ}\right)}}{S_{\left(\text{τ}\right)}}={\left(c\text{τ}\right)}^{H}$$

The value of *H* is obtained by fitting the equation $log{\left(R/S\right)}_{n}=a+H\times \text{log}\left(n\right)$ , which can be used to determine whether the time series {NDVI(τ)} is completely random or persistent. According to previous studies, the value of the Hurst exponent, ranging from 0 to 1, is classified into three types. When 0.5 < *H*< 1, the trend of change in NDVI in the future will be consistent with that in the past. When *H* = 0.5, the NDVI time series is random and there is no long-term correlation. When 0 < *H*< 0.5, the trend in the future will be the opposite of that in the past.

#### 2.3.3 Sequential Mann–Kendall test

The sequential Mann–Kendall (SQMK) test was used to identify the change point of NDVI of wetland vegetation and four climatic factors (PRE, TMP, TMX, and TMN) from 1982 to 2020. The SQMK test, developed by Sneyers (1991), is used to detect abrupt change points in long-term data series. The steps of the SQMK test are the following:

1) For a time, series with *n* sample sizes *X {X*_1_, *X*_2_,*…, X_n_}*, a rank sequence is constructed as follows:


$$S_{k}=\sum_{i=1}^{k}R_{i\;}(k=2,3,\cdots ,n)$$

where *R_i_* is the cumulative number of samples when $X_{i}>X_{j}\left(1\leq j\leq i\right)$ .

2) Under the assumption of random independence of time series, the statistics are defined as follows:


$$UF_{k}=\frac{S_{k}-E\left(S_{k}\right)}{\sqrt{Var\left(S_{k}\right)}}\;\left(k=1,2,\cdots ,n\right)$$

where *UF_1 =_* 0, *E*(*S_k_*) and *Var*(*S_k_*) are the mean and variance of the cumulative number *S_k_*, respectively. When *X{X*_1_, *X*_2_,*…, X_n_}* is independent of the others and has the same continuous distribution, $E\left(S_{k}\right)=\frac{n\left(n-1\right)}{4}$ , and $Var\left(S_{k}\right)=\frac{n\left(n-1\right)\left(2n+5\right)}{72}$ . For a given significance level α, *UF_k_* > *UF*_α_ indicates a significant trend change in the sequence of *X*.

3) The time series data are arranged in reverse order, and the above calculation is repeated to obtain *UB_k_*, indicated as follows:


$$UB_{k}=-UF_{k}$$

4) When the significance level α is set to 0.05, as in this study, the value of *U_1-_*_α_ *_/2_* is ± 1.96 (Wang R. et al., 2020). The intersection points of *UF* and *UB* curves indicate the abrupt change year in a time series trend (Mosmann et al., 2004).

#### 2.3.4 Correlation analysis method at the scale of pixels

A correlation coefficient method at the scale of pixels was used to evaluate correlations between NDVI and climatic factors (TMP, TMX, TMN, and PRE) from 1982 to 2020. The method was processed in ArcGIS based on the ArcPy tool, which can investigate possible effects of climate change on NDVI. The formula is as follows:


$$r_{xy}=\frac{\sum_{i=1}^{n}\left(x_{i}-\bar{x}\right)\left(y_{i}-\bar{y}\right)}{\sqrt{\sum_{i=1}^{n}{\left(x_{i}-\bar{x}\right)}^{2}}\sqrt{\sum_{i=1}^{n}{\left(y_{i}-\bar{y}\right)}^{2}}}$$

where *r_xy_* is the correlation coefficient between variables *x* and *y*, ranging from −1 to 1, *n* is the number of years, *x_i_* is the value of the NDVI for year *i*, *y_i_* is the value of the climatic factors (TMP, TMX, TMN, and PRE) for year *i*, and $\bar{x}$ and $\bar{y}$ are the averaged NDVI and the mean of the climatic factors, respectively, which were obtained from the averages of all pixels assigned to wetlands (Chen et al., 2018). When *r_xy_* > 0, the NDVI of wetland vegetation and the climatic factors were positively correlated. When *r_xy_* < 0, the two variables were negatively correlated.

Moreover, the correlations of NDVI with climatic factors were classified into four major categories: significant positive correlations (*p* < 0.05), nonsignificant positive correlations (*p* > 0.05), significant negative correlations (*p* < 0.05), and nonsignificant negative correlations (*p* > 0.05). In addition, we considered the lag effects of precipitation, so we calculated the correlation coefficients between the NDVI of the current year and the precipitation of the previous year and the previous 2 years.

#### 2.3.5 Partial correlation analysis

Partial correlation analysis was used to calculate the partial correlation coefficients between PRE, TMP, and NDVI, respectively, to examine interaction term between temperature and precipitation. When the dependent variable is correlated with two or more independent variables, partial correlation analysis is used to investigate the correlation between one independent variable and the dependent variable by excluding the influence of another independent variable (Cao et al., 2014).

The formula is as follows:


$$r_{xy.z}=\frac{r_{xy}-r_{xz}r_{yz}}{\sqrt{\left(1-r_{xz}^{2}\right)\left(1-r_{yz}^{2}\right)}}$$

where *r_xy_*, *r_xz_*, and *r_yz_* is the correlation coefficient between variables *x* and *y*, *x* and *z*, and *y* and *z*, respectively, ranging from −1 to 1, *r_xy.z_* is the partial correlation coefficient between *r_xy_* and *r_xz_* fixed *r_yz_*. The value of the partial correlation coefficient ranges from +1 to −1.

## 3 Results

### 3.1 Spatiotemporal changes in the NDVI of wetland in the ARB

**Figure 2:**
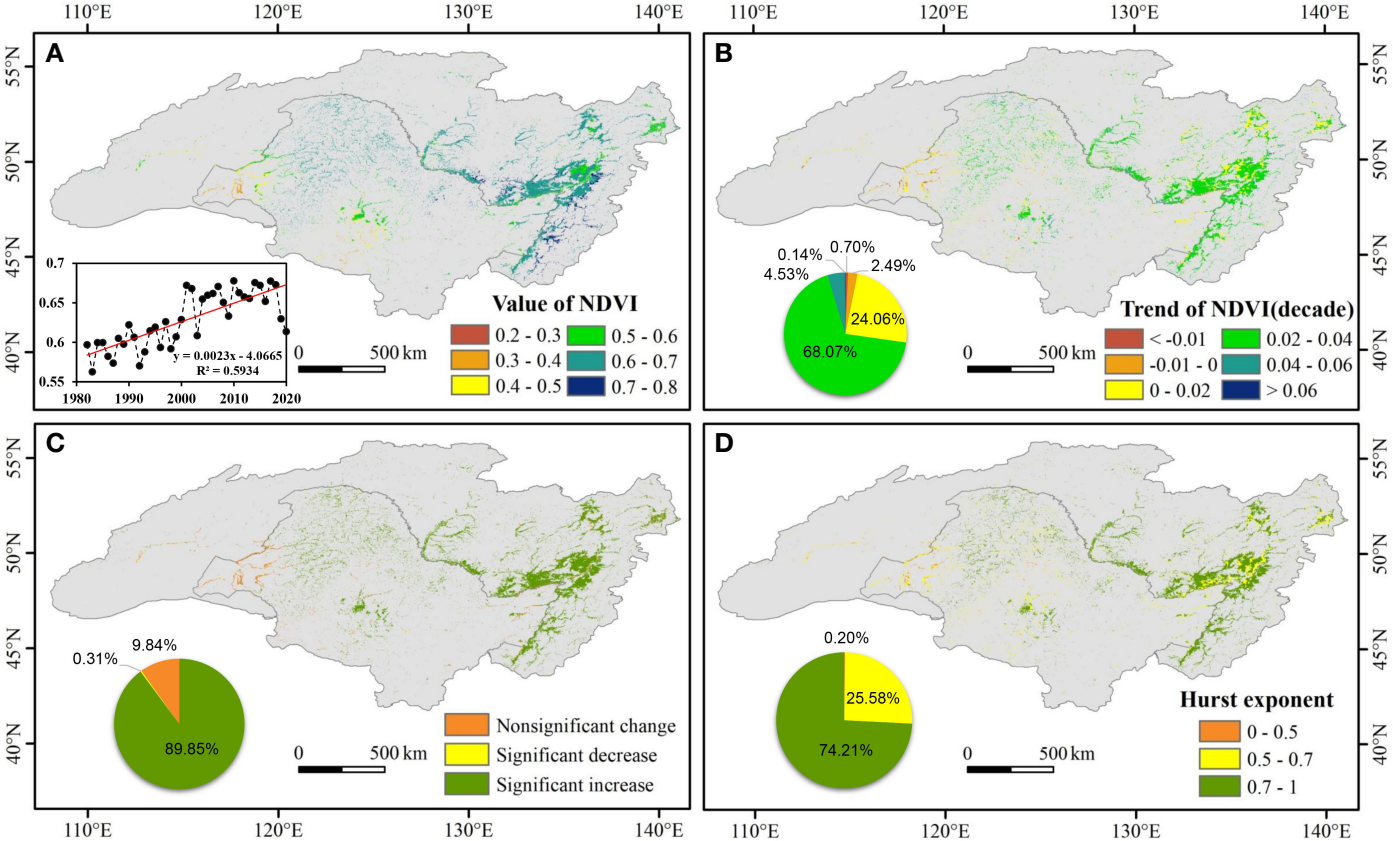
Spatial pattern of wetland normalized difference vegetation index (NDVI) in the Amur River basin from 1982 to 2020. **(A)** Spatial distribution of the average NDVI of wetland during the annual growing season, inset shows the temporal changes of NDVI; **(B)** spatial pattern in temporal trend (per decade) of NDVI; **(C)** spatial distribution of trend types; **(D)** spatial pattern in Hurst exponent of NDVI. The pie charts illustrate the area percentage of NDVI trends and sustainable characteristics.

At the basin scale, the mean NDVI of wetland in the growing season showed a significantly increasing trend (*p* < 0.01) from 1982 to 2020 with a growth rate of 0.023 per decade, although there was a minimum value in 2003 (**Figure 2A**). At the pixel scale, relatively large the NDVI of wetland values were mainly distributed in the eastern and central parts of the ARB, such as the Greater and Lesser Khingan mountains and the eastern Russian side. Relatively small NDVI values were mainly distributed in the southwestern part of the basin.

According to Sen+Mann–Kendall analysis, variations in the NDVI of wetland from 1982 to 2020 in the ARB had apparent spatial heterogeneity (**Figure 2B**). With the Greater Khingan Mountains as a border, the NDVI of wetland primarily decreased in the relatively dry western region but increased in the relatively wet eastern region. In the ARB, 89.85% of pixels experienced significant increases (*p* < 0.05) in NDVI in the growing season (**Figure 2C**).

The mean value of the NDVI Hurst index for wetland vegetation was 0.71. As shown in **Figure 2D**, the Hurst index exceeded 0.5 in much of the ARB. Pixels with Hurst index values between 0.5 and 0.7 and greater than 0.7 accounted for 25.58% and 74.21%, respectively.

On the Chinese side of the ARB, the average the NDVI of wetland was 0.59, the trend of increase in average NDVI was 0.02 per decade, and the average Hurst index was 0.68. However, on the Russian side, the average the NDVI of wetland was 0.65, the trend of increase in average NDVI was 0.03 per decade, and the average Hurst index was 0.73. Thus, in the comparative analysis of Chinese and Russian sides, values on the Russian side were all higher than those on the Chinese side.

**Figure 3:**
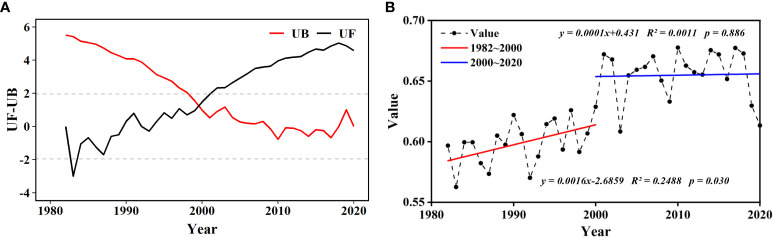
Abrupt and temporal changes in wetland normalized difference vegetation index (NDVI) in the Amur River basin (ARB) from 1982 to 2020: **(A)** abrupt change of the NDVI of wetland using SQMK test. Dotted horizontal straight lines indicate the lower limit and upper limit of 95% confidence interval. **(B)** temporal changes of the NDVI of wetland in the ARB during 1982–2000 and 2000–2020. *p* < 0.05 and *p* < 0.01 indicate significance at the 95% and 99% levels.

According to the SQMK test, the NDVI of wetland values in the ARB from 1982 to 2020 changed abruptly in 2000 (**Figure 3A**). During 1982–2000, the average NDVI fluctuated between 0.55 and 0.65, and the trend in the NDVI of wetland in the ARB was clearly one of increase. However, during 2000–2020, although the average NDVI fluctuated between 0.60 and 0.70, no significant trend of increase was observed (**Figure 3B**).

### 3.2 Spatiotemporal change in climatic factors in the Amur River basin

**Figure 4:**
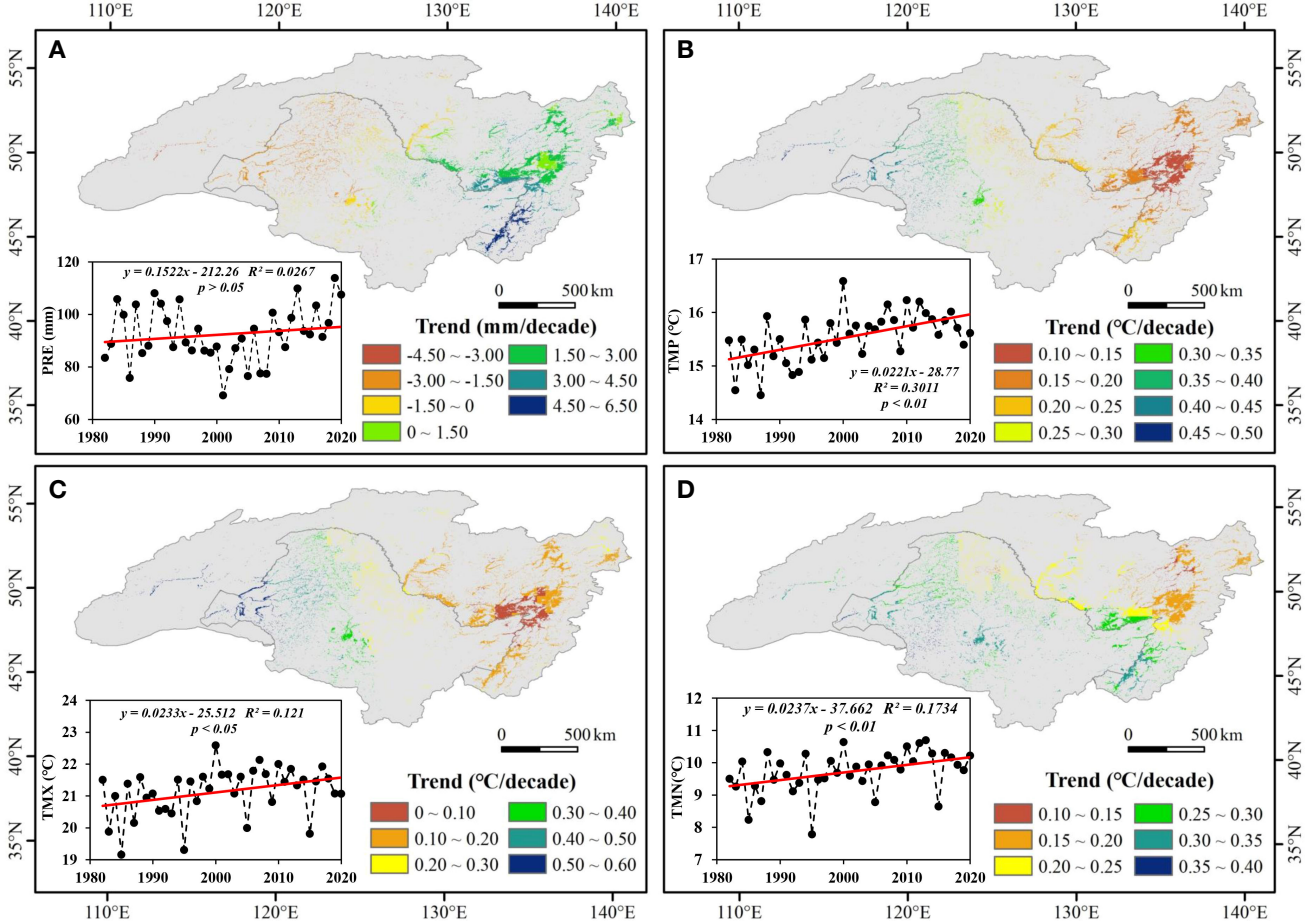
Spatiotemporal changes in **(A)** precipitation (PRE), **(B)** daily mean temperature (TMP), **(C)** monthly average daily maximum temperature (TMX), and **(D)** monthly average daily minimum temperature (TMN) in the Amur River basin from 1982 to 2020. *p* < 0.05 and *p* < 0.01 indicate significance at the 95% and 99% levels.

From 1982 to 2020, increasing trend of PRE, TMP, TMX, and TMN were observed at different levels (**Figure 4**). The PRE had a growth rate of 1.5 mm per decade, and temperature (TMP, TMX, and TMN) increased significantly with a growth rate of 0.2°C per decade. In terms of spatial changes, PRE showed a downward trend in the west, mainly in the Songnen Plain, but an upward trend in the east, mainly in the Sanjiang Plain, with the reversal line at approximately 130°E. The temperature increase was higher in the western ARB than in the eastern ARB. On the Chinese side, PRE showed a downward trend, whereas on the Russian side, it showed an upward trend, indicating clear spatial heterogeneity. The magnitude of temperature increase in China was significantly higher than that in Russia.

**Figure 5:**
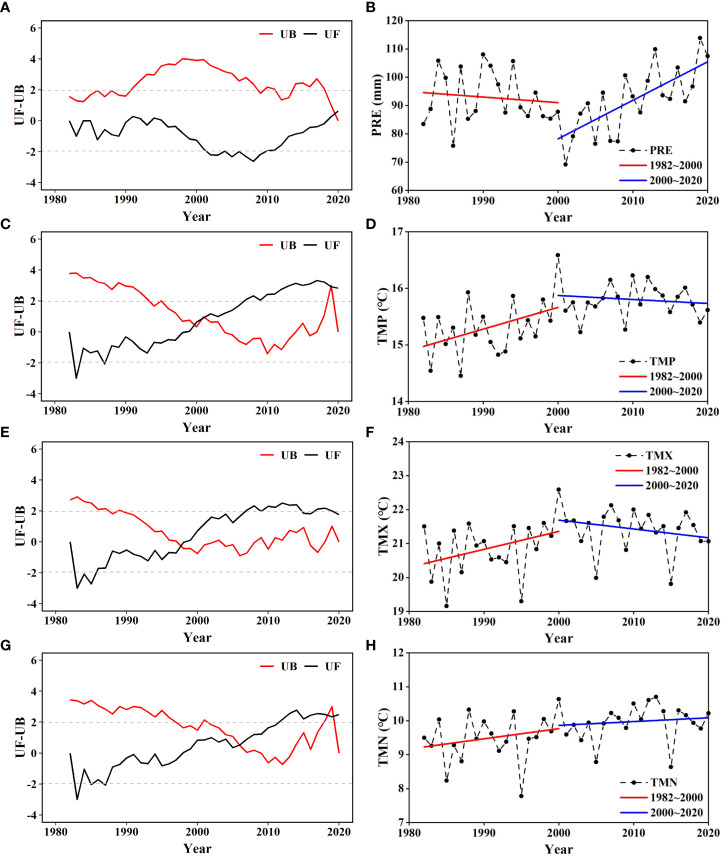
Abrupt change in **(A, B)** precipitation (PRE), **(C, D)** daily mean temperature (TMP), **(E, F)** monthly average daily maximum temperature (TMX), and **(G, H)** monthly average daily minimum temperature (TMN) in the Amur River basin.

From 1982 to 2020, an SQMK test revealed the abrupt change point in PRE occurred in 2019 (**Figure 5**). During the 4 decades, two break points in TMP occurred in 2000 and 2019, one break point in TMX occurred in 1998, and three break points in TMN occurred in 2006, 2018, and 2019.

Because abrupt changes in both NDVI and TMP occurred in 2000, the interannual variation in each climatic factor was analyzed in two stages separated by 2000 (**Figure 5**). Precipitation showed an overall decreasing trend from 1982 to 2000 but an increasing trend from 2000 to 2020. Daily mean temperature exhibited an increasing trend from 1982 to 2000 but a decreasing trend from 2000 to 2020. The trends in TMX were almost the same as those in TMP, but the trend in TMN was continuous increase in both periods.

**Figure 6:**
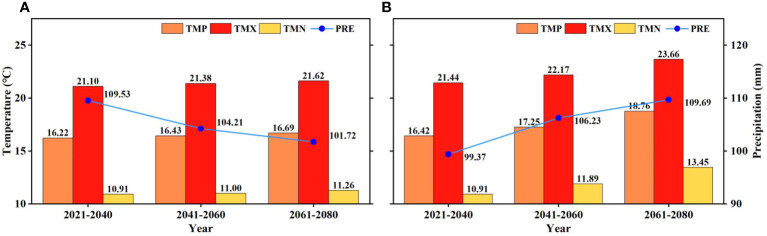
Future climate under **(A)** SSP126 and **(B)** SSP585 scenarios in the growing season from 2021 to 2080 in the Amur River basin.

According to the CNRM-ESM2-1 model, the climate will become warmer and drier under the SSP126 scenario (low-emission scenario) in the ARB in the growing season from 2021 to 2080, but under the SSP585 scenario (high-emission scenario), it will become warmer and wetter (**Figure 6**). In addition, the growth rate in TMN will be higher than that in TMX under the SSP585 scenario, whereas the difference will be not obvious under the SSP126 scenario.

### 3.3 Spatiotemporal patterns of correlations between the NDVI of wetland and climatic factors

**Table 1** shows the correlations between the NDVI of wetland and the four climatic factors throughout the ARB. In terms of temporal correlation, the NDVI of wetland in the ARB was significantly and positively correlated with TMP, TMX, and TMN (*p* < 0.05) and negatively correlated with PRE (*p* > 0.05). The highest correlation was with TMP (*r* = 0.701). On both Chinese and Russian sides of the ARB, the NDVI of wetland was significantly and positively correlated with temperature (*p* < 0.05) but negatively correlated with PRE. In addition, partial correlation analysis revealed that TMP remained significantly and positively correlated with NDVI, while PRE was negatively correlated with NDVI except for Chinese side. In terms of the lag effects of precipitation and NDVI, the correlation coefficients between the NDVI of the current year and the precipitation of the previous year and the previous 2 years were −0.7 (*p* > 0.05) and 0.157 (*p* > 0.05), respectively.

**Table 1:** Pearson correlation coefficients between NDVI and temperature (daily mean temperature, TMP; monthly average daily maximum temperature, TMX; monthly average daily minimum temperature, TMN) and precipitation (PRE) and partial correlation coefficients between NDVI and TMP and PRE in the Amur River basin and different nations during 1982–2020.

Study area	Pearson correlation coefficients				Partial correlation coefficients	
	R_NDVI_PRE_	R_NDVI_TMP_	R_NDVI_TMX_	R_NDVI_TMN_	R_NDVI_PRE_	R_NDVI_TMP_
ARB	−0.12	0.701**	0.423**	0.386*	−0.016	0.648**
Chinese side	−0.224	0.728**	0.413**	0.487**	0.018	0.655**
Russian side	−0.052	0.608**	0.350*	0.340*	−0.044	0.556**

* and ** represent significance at the 0.05 level and the 0.01 level, respectively.

**Figure 7:**
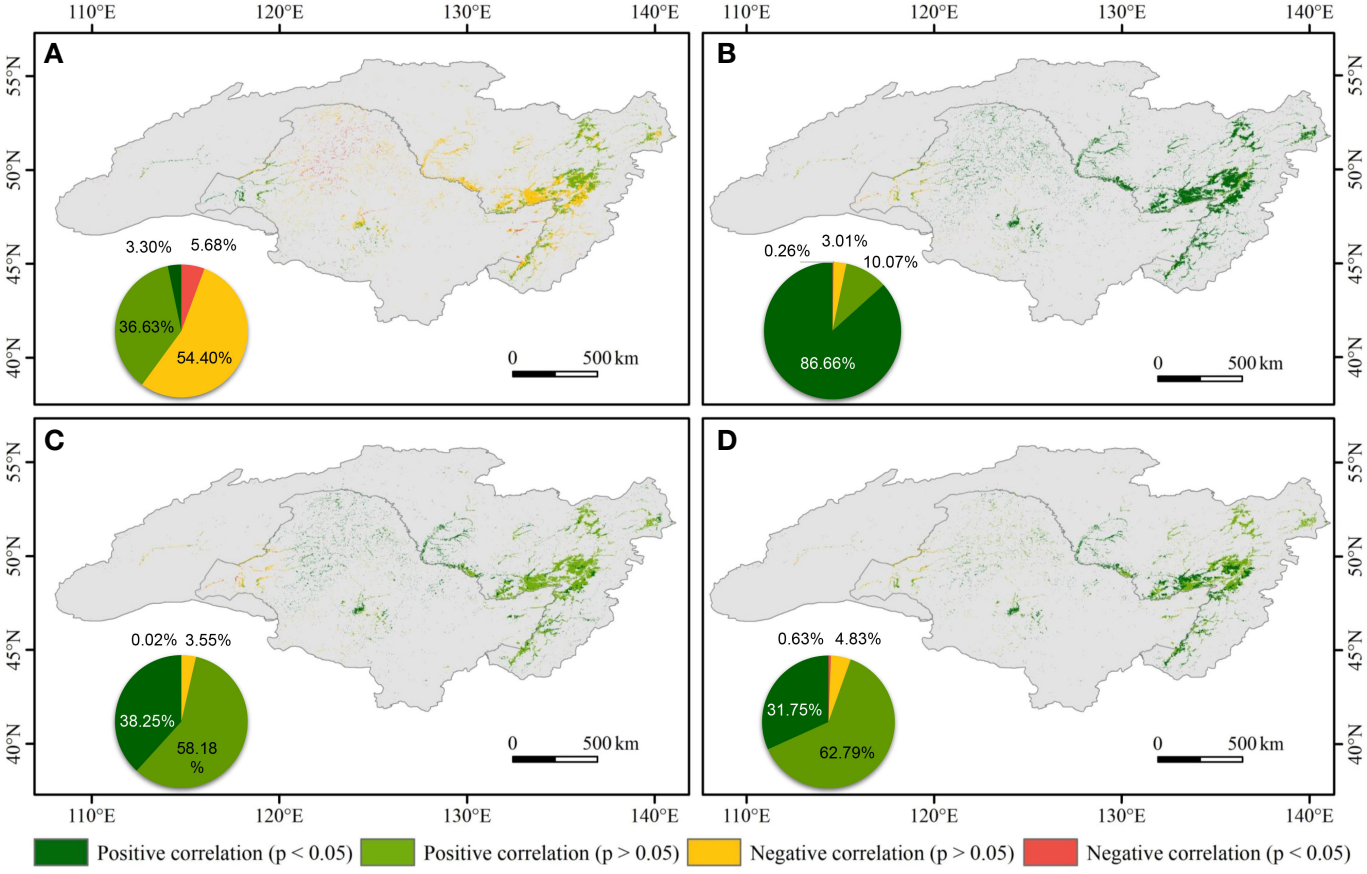
Spatial patterns of correlations between normalized difference vegetation index (NDVI) and **(A)** precipitation (PRE), **(B)** daily mean temperature (TMP), **(C)** monthly average daily maximum temperature (TMX), and **(D)** monthly average daily minimum temperature (TMN) in the Amur River basin during 1982–2020. The pie charts illustrate the area percentage of different spatial patterns of the correlations.

Spatially, there was a weak negative correlation between the NDVI of wetland and PRE in the central ARB, whilst the NDVI of wetland was positively correlated with PRE in the eastern and western ARB (**Figure 7**). At the pixel scale, 86.66% of the ARB showed significant positive correlations between the NDVI of wetland and TMP (*p* < 0.05), distributed throughout the ARB except in the west. Positive correlations between NDVI and TMX and between NDVI and TMN were mainly in the central and eastern ARB. On the Chinese side of the ARB, correlations between the NDVI of wetland and PRE were primarily negative, whereas on the Russian side, there were both positive and negative correlations between the NDVI of wetland and PRE. The NDVI of wetland was significantly and positively correlated with temperature on both Chinese and Russian sides of the ARB.

According to the analysis of the two stages with 2000 as the node, correlations between the NDVI of wetland and the four climatic factors varied in different time periods and different countries (**Table 2**). There was a weak asymmetric effect of diurnal warming on wetland vegetation in the ARB during 2000–2020, which means Pearson correlation coefficients between NDVI and TMX and TMN are smaller than 0.2 and significances are greater than the 0.05 level. The NDVI of wetland was positively correlated with daily maximum temperature but negatively correlated with daily minimum temperature. On Chinese and Russian sides of the ARB during the two periods, the NDVI of wetland was primarily negatively correlated with PRE and significantly and positively correlated with TMP (*p*< 0.01). Nevertheless, on the Russian side, NDVI and PRE were positively correlated during 1982–2000, and NDVI and TMP were negatively correlated during 2000–2020.

**Table 2:** Pearson correlation coefficients between normalized difference vegetation index (NDVI) and four climatic factors in different regions during two periods.

Periods	Study area	PRE	TMP	TMX	TMN
1982–2000	ARB	0.178	0.642^**^	0.302	0.291
	Chinese side	−0.009	0.602^**^	0.325	0.245
	Russian side	0.178	0.622^**^	0.27	0.291
2000–2020	ARB	−0.354	0.351	0.14	−0.012
	Chinese side	−0.168	0.259	0.07	0.011
	Russian side	−0.391	−0.011	0.15	0.319

* and ** represent significance at the 0.05 level and the 0.01 level, respectively.

**Figure 8:**
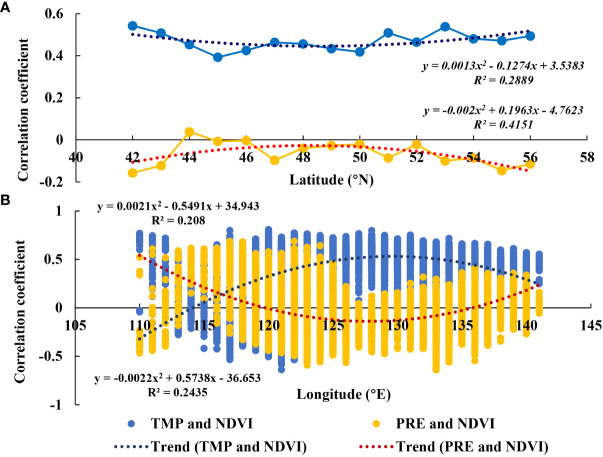
Changes in correlation coefficients between normalized difference vegetation index (NDVI) and temperature and precipitation (PRE) in **(A)** latitudinal and **(B)** longitudinal directions.

Pronounced latitudinal and longitudinal zonal variability was observed among correlation coefficients (**Figure 8**). With an increase in latitude, correlation coefficients between NDVI and TMP increased before decreasing, whereas those between NDVI and PRE decreased before increasing. However, in the longitudinal direction, trends in correlation coefficients between NDVI and TMP and PRE were opposite to those in the latitudinal direction. Notably, 49°N and 130°E were apparent points for the change between increasing and decreasing correlation coefficients.

## 4 Discussion

### 4.1 The NDVI of wetland dynamics and its relations with climatic factors

The NDVI of wetland showed a significant upward trend during 1982–2020, which was primarily attributed to increases in temperature and precipitation. A warm and humid environment is conducive to the growth of wetland vegetation (Burkett and Kusler, 2000). Higher temperature can increase greater photosynthetic intensity, which played an important role for the growth of vegetation (Sharma et al., 2020). The percentage increase in the NDVI trend and the Hurst index indicated good overall growth of wetland vegetation during the four decades examined in this study. In addition, according to the results of Hurst exponent, the predicted future change in the NDVI of wetland trend in the ARB was consistent with that of the past trend (**Figure 2D**). The minimum point of the NDVI of wetland occurred in 2003 but temperature, affected vegetation growth strongly, was not the lowest value, and precipitation was not strongly correlated with wetland vegetation. However, the minimum point of precipitation occurred in 2001, so we considered whether the minimum NDVI in 2003 could be caused by the lagging effect of precipitation and spring drought led to a large reduction. The correlation coefficients were −0.7 (*p* > 0.05) and 0.157 (*p* > 0.05), respectively, which indicated that there were lag effects of PRE on the NDVI of wetland in the ARB.

According to the SQMK test, average the NDVI of wetland in the ARB increased after 2000. The increase in average NDVI was probably attributed to recognition of the importance of wetlands beginning in the late 1990s and China’s promulgation of the China Action Plan for Wetland Conservation in 2000 (Chen et al., 2018). In addition, Northeast China responded positively to the national policy. For example, Heilongjiang Province promulgated *the Decision on Wetland Protection* in 1998 and the *Heilongjiang Wetland Protection Regulations* in 2003 (Cui, 2006). However, the rate of increase in the NDVI of wetland slowed after 2000, which was likely associated with the decline in TMP during 2000–2020. The global warming interruption from 1999 onward resulted in a weak cooling trend in TMP (Fyfe et al., 2013; Karl et al., 2015), also called the warming interval (Li et al., 2015). Because the abrupt changes in both NDVI and TMP occurred in 2000, it also confirmed that the NDVI of wetland in the ARB was mostly influenced by TMP. Increases in temperature lead to increases in photosynthetic intensity of plants (Sharma et al., 2020), thus promoting the growth of vegetation in the growing season, according to Yang et al. (2020). In further analysis of the relations between NDVI and daytime and nighttime temperatures in the ARB, weak asymmetric effects of diurnal warming on wetland vegetation were detected during 2000–2020. The growing season NDVI of wetland vegetation was positively correlated with TMX and negatively correlated with TMN, which is consistent with the Peng et al. (2013). Wetland vegetation perform photosynthesis during the daytime, and increasing TMX can enhance photosynthetic enzyme activity and carbon dioxide concentration, thus promoting vegetation growth (Lucht et al., 2002; Beer et al., 2010). Respiration in plants occurs mainly at night, via enhanced autotrophic respiration and produced a compensatory stimulation of photosynthesis the next day (Wan et al., 2009), which could partly explain the positive correlation between NDVI and TMN.

### 4.2 Wetland vegetation changes in response to climate change at multiple scales

The growing season trends in the NDVI of wetland and its responses to climate change on Chinese and Russian sides of the ARB had obvious spatial heterogeneity. The NDVI of wetland was primarily negatively correlated with PRE and significantly positively correlated with TMP on the Chinese and Russian sides. Nevertheless, PRE had a positive effect on the NDVI of wetland in Russia during 1982–2000, possibly because adequate water promoted wetland vegetation growth. Russia is geographically closer to the sea than China; affected by the position of sea and land, there was more precipitation in Russia, especially in the east of Russia (**Figure 4**). During 2000–2020, the NDVI of wetland and TMP were negatively correlated on the Russian side, which might be associated with permafrost degradation under a warming climate. Permafrost covers 60% of the entire basin (Avis et al., 2011), and many wetlands in cold regions live in symbiosis with permafrost. Permafrost prevents precipitation and runoff from percolating into the ground, and excessive surface moisture inhibits aerobic bacterial activity, and promotes peat accumulation, which contributes to the growth of wetland vegetation (Jin et al., 2008). China is located in a mid-temperate region with higher temperature, which is the strong climatic factor affecting vegetation growth. However, the growth of wetland vegetation on the Russian side was better than that on the Chinese side. This discrepancy could be attributed to not only the influence of climate but also the different intensity of human activity (Mao et al., 2021). In addition, over-cultivation of rice in northeastern China, an important grain production base, competes with wetlands for water (Zou et al., 2018). Surface water is reintroduced from the subsurface to irrigate farmland in some areas due to excessive oxidative enrichment, resulting in a decline in wetland water levels (Zhou et al., 2021).

**Figure 9:**
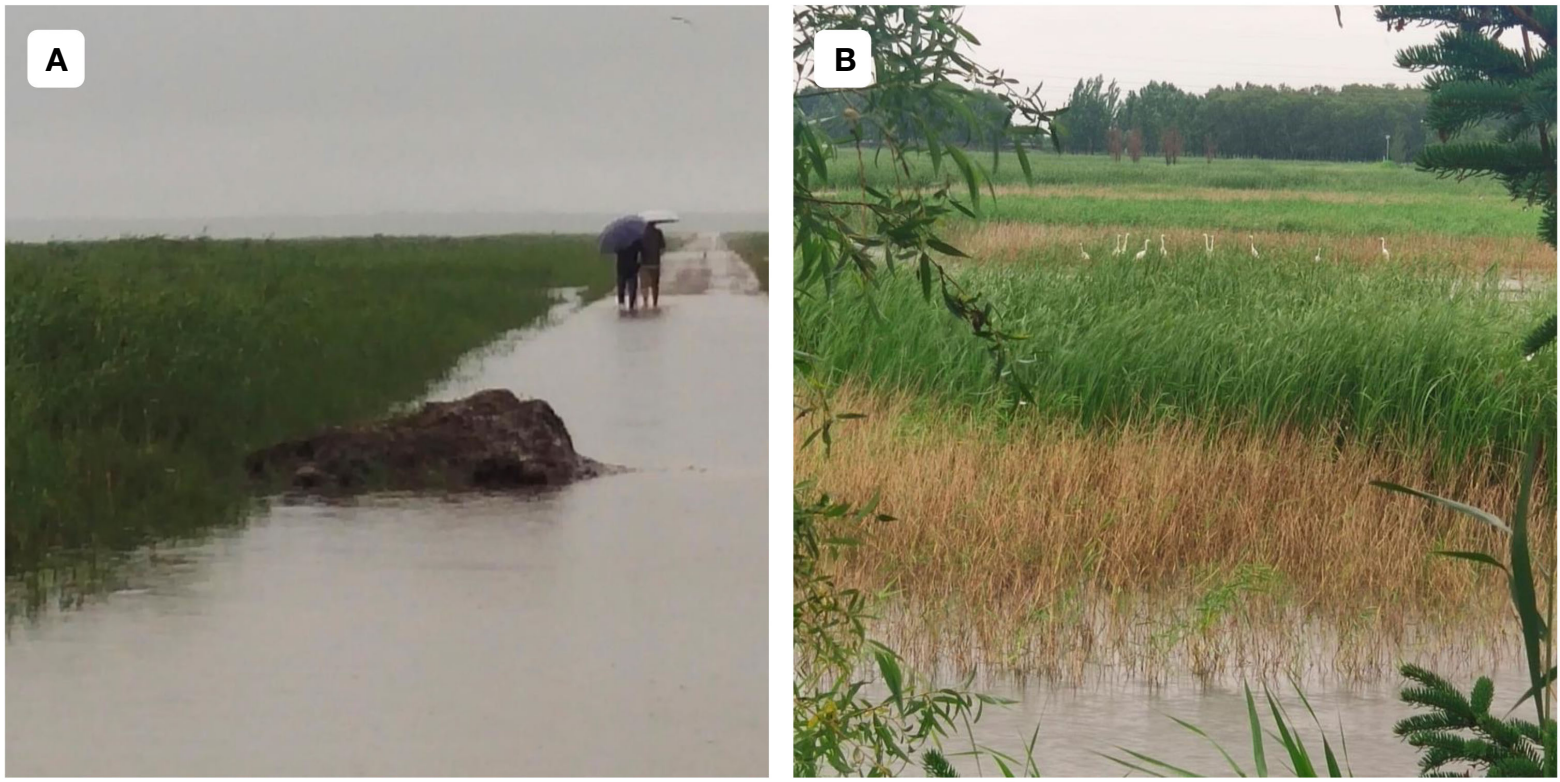
Excessive PRE condition in the ARB **(A)** and the brown parts indicate the death of the wetland vegetation **(B)**.

Notably, correlation coefficients between NDVI and PRE and TMP in the ARB were zonal in latitudinal and longitudinal directions, and 49°N and 130°E were the apparent turning points between increasing and decreasing correlation coefficients. In the latitudinal direction, 49°N is roughly consistent with the boundary between mid-temperate and frigid-temperate zones (Wang et al., 2021). In the frigid-temperate zone, relatively high temperatures are associated with increases in effective soil nitrogen, which promote wetland vegetation growth by increasing photosynthetic intensity of the vegetation (Amundson et al., 2003). In addition, increases in rainfall are associated with an increase in cloud cover and a decrease in sunlight, which may limit vegetation growth (Mao et al., 2012). In the longitudinal direction, 130°E is roughly consistent with the boundary between humid and semi-humid zones (Zhang et al., 2019). The eastern part of the ARB is primarily in the humid region, whereas the western part is in the semi-humid region. In semi-humid areas, wetland vegetation is hydrophilic, and PRE is the main factor influencing its growth (Li et al., 2013). However, with an increase in longitude in the humid area, excessive PRE leads to the drowning and death of some wetland vegetation (**Figure 9**), including Phragmites australis and Scirpus triqueter, which are the dominant wetland vegetation in the ARB (Xie et al., 2008). Except for the drowning of wetland vegetation, the increase of clouds and the reduction of sunshine caused by excessive PRE inhibits the growth of wetland vegetation (Yang et al., 2020). Therefore, correlation coefficients between PRE and NDVI decreased, and TMP was a major factor affecting vegetation growth.

### 4.3 Projection of future climate change effects on wetland vegetation in the Amur River basin

The warming and wetting in the ARB during the 4 decades of this study are consistent with results of previous researches (Yu et al., 2013; Chu et al., 2019). According to the observations and the analysis of data by various organizations, global climate change is dramatic (IPCC, 2007; Potter et al., 2017). In the future, the climate warming trend will continue in the Northern Hemisphere (Salinger, 2005). In the ARB, temperature and precipitation are predicted to increase by the end of the 21st century under the SSP585 scenario (Yang et al., 2021), and the area of marshland is predicted to increase on the Songnen and Sanjiang plains (Chen et al., 2018). Compared with Canada in the same latitudes, the future warmer and wetter climate will favor increased wetland abundance in the western prairies (Zhang et al., 2021). Future climate change (i.e., warming, variation in precipitation) will affect the growth of wetland vegetation through photosynthesis, respiration and transpiration. In order to predict future climate change conditions, CMIP6 developed different emission scenarios to simulate different possible socioeconomic developments (O'Neill et al., 2016). SSP–RCP was widely used in regional climate change prediction (Yang et al., 2020; Ren et al., 2022). To further explore the future growth of wetland vegetation in the entire ARB, the NDVI of wetland from 2021 to 2080 was predicted selecting SSP1–RCP2.6 (SSP126) and SSP5–RCP8.5 (SSP585).

Under the SSP126 scenario, warming in the future will increase the NDVI of wetland (Sharma et al., 2020), and the relatively dry but still humid conditions will encourage growth of wetland vegetation in the future (Xie et al., 2008). Under the high-emission scenario, the NDVI of wetland will also show an upward trend from 2021 to 2080, because temperature will have a greater effect on the growth of wetland vegetation than that of precipitation in the ARB (**Table 1**). Notably, in the humid area, an increase of precipitation will cause a decrease in NDVI (Mao et al., 2012). In terms of diurnal temperature, the negative effect of the rapid rise in TMN on the NDVI of wetland will gradually increase and might even exceed the positive effect of TMX in the future.

## 5 Conclusions

Based on NDVI and CRU data, this study investigated spatiotemporal changes in the NDVI of wetland and its responses to climate change in the ARB during 1982–2020 in different countries, geographic gradients, and time periods. From 1982 to 2020, the NDVI of wetland increased significantly (*p* < 0.01) at the rate of 0.023 per decade, but the increasing rate gradually slowed. Approximately 89.85% of the total basin experienced significant increases in the NDVI of wetland in the growing season (*p* < 0.05). Correlation analysis and SQMK method both indicated that the NDVI of wetland responded most strongly to TMP. Asymmetric diurnal warming was detected during 2000–2020 by comparing the trend in TMX with that in TMN. The NDVI of wetland was positively correlated with TMX (*r* = 0.14, *p* > 0.05), but it was negatively correlated with TMN (*r* = −0.012, *p* > 0.05). The influence of PRE on wetland was weak and not significant. In addition, correlations between the NDVI of wetland and climatic factors were zonal in latitudinal and longitudinal directions. Under the SSP126 and SSP585 scenarios, the climate will become warmer, and the NDVI of wetland in the ARB is predicted to increase until 2080. Note that the negative effect of the rapid rise in TMN on the NDVI of wetland will gradually increase and might even exceed the positive effect of TMX in the future. The climatic factors affecting wetland NDVI are not only temperature and precipitation, but also solar radiation and wind speed et al. In addition to climate factors, human activities also have a great impact on wetlands, and human factors were rarely discussed in this study. The findings of this study are expected to provide support for wetland conservation and sustainable management in the ARB.

## Data availability statement

The original contributions presented in the study are included in the article/supplementary material. Further inquiries can be directed to the corresponding authors.

## Author contributions

ZX: Data curation, Investigation, Methodology, Writing – original draft, Writing – review & editing. DM: Conceptualization, Funding acquisition, Software, Supervision, Writing – review & editing. XL: Conceptualization, Funding acquisition, Methodology, Writing – review & editing. LL: Data curation, Formal Analysis, Methodology, Writing – review & editing. ZW: Conceptualization, Resources, Supervision, Writing – review & editing.
